# To clip or not to clip? A review of precise risk assessment in the contemporary era of transcatheter edge-to-edge mitral and tricuspid valve repair

**DOI:** 10.3389/fcvm.2025.1693291

**Published:** 2025-11-14

**Authors:** Felix Ausbuettel, Carlo-Federico Fichera

**Affiliations:** 1 Department of Cardiology, University Hospital Marburg, Marburg, Germany; 2 County Hospital Loerrach, Department of Cardiology, Loerrach, Germany

**Keywords:** M-TEER, T-TEER, mortality prediction, transcatheter edge-to-edge repair, mitraclip, triclip, PASCAL

## Abstract

Transcatheter edge-to-edge mitral and tricuspid valve repair (M-TEER, T-TEER) have emerged as meaningful treatment modalities among patients at high surgical risk suffering from valvular heart disease. While previous research has shown that optimal patient selection is crucial for treatment outcomes, recent studies have identified a multitude of factors that independently influence mortality. Although these findings can significantly support clinical decision-making, the large number of available studies renders an overview of this topic challenging. In this review, we provide a comprehensive overview of the currently identified factors associated with increased mortality after TEER. We also summarize the current evidence on published risk scores that stratify mortality risk after M-TEER and T-TEER. We aimed to provide clinical decision-making support for optimal patient selection and referral to TEER and to identify remaining gaps in evidence.

## Graphical Abstract

**Figure:**
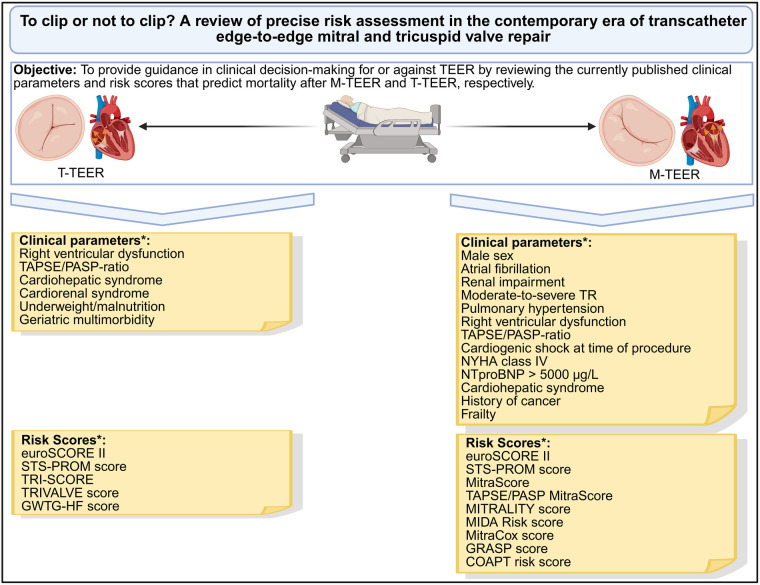
*Clinical parameters or risk scores that were independently associated with mortality after M-TEER or T-TEER, respectively. M-TEER, transcatheter edge-to-edge mitral valve repair; NYHA, New-York-Heart-Association; PASP, pulmonary artery systolic pressure; TAPSE, tricuspid annular plane systolic excursion; TEER, transcatheter edge-to-edge repair; TR, tricuspid valve regurgitation; T-TEER, transcatheter edge-to-edge tricuspid valve repair. Created in BioRender. Ausbüttel, F. (2025) https://BioRender.com/yu30ckm.

## Introduction

In Western industrialized nations, mitral regurgitation (MR) is the most common form of valvular heart disease (VHD) in patients aged >75 years (1). Among patients suffering from congestive heart failure, severe tricuspid regurgitation (TR) serves as a surrogate parameter for advanced disease stage with a consecutive increase in mortality (2, 3). Since both VHDs are particularly common among elderly patients, whose increased age and higher rate of comorbidities lead to increased perioperative risk, these patients are frequently deemed ineligible candidates for surgical valve repair (4). Transcatheter edge-to-edge repair (TEER) was developed to ameliorate the overall therapeutic prognosis of this particular cohort of patients (5, 6). Both transcatheter edge-to-edge mitral valve repair (M-TEER) and transcatheter edge-to-edge tricuspid valve repair (T-TEER) have proven to be safe and efficacious treatment modalities for patients at high surgical risk who remain symptomatic despite optimal medical therapy (OMT) for VHD (7–12).

Owing to ongoing demographic changes, a further increase in procedure numbers are expected (4, 13). The lessons learned from the existing evidence on TEER revealed that optimal screening and referral of suitable candidates for TEER remain crucial to achieve favorable treatment outcomes (14, 15), although both M-TEER and T-TEER were proven successful in alleviating the symptom burden of VHD, quality of life and hospitalization rates in these distinct cohorts of patients (5, 6, 9, 12).

We therefore aimed to provide a comprehensive review of the existing evidence concerning the determinants of mortality in current “real-world“ patients who have undergone M-TEER or T-TEER. For this purpose, research was conducted into clinical parameters with a proven significant influence on mortality, as well as risk scores for predicting mortality, whose accuracy was presented using the area under the curve (AUC) value or c-statistics as reported in the original publication, respectively.

### Risk assessment prior to intervention

Previous studies have identified a variety of independent mortality predictors for both procedures, which have partly been summarized in scores for predicting mortality. These are presented separately for both procedures:

### Transcatheter edge-to-edge mitral valve repair (M-TEER)

#### Clinical parameters with significant effects on mortality after M-TEER

One of the clinical predictors of mortality was male sex (16), which was associated with a higher rate of complicating comorbidities (17). Further comorbidities that were associated with significantly increased mortality included concomitant atrial fibrillation (AF) (18), renal impairment (19), moderate to severe tricuspid regurgitation (TR) (20, 21), pulmonary hypertension (22) and right ventricular dysfunction (RVD) (23, 24). For the measurement of RVD, the ratio of the echocardiographic values of the tricuspid annular plane systolic excursion (TAPSE) and the pulmonary arterial systolic pressure (PASP) was established, which defined the uncoupling between the right ventricle (RV) and the pulmonary artery (PA) as a marker of significantly worsened survival (25). With respect to left ventricular ejection fraction (LVEF) as a further central echocardiographic parameter, a wide range of studies revealed inconsistent results with no clear observable effect on mortality (26). However, the performance of M-TEER in patients with reduced LVEF due to cardiogenic shock was associated with an increased mortality rate (27). In the context of advanced heart failure, heart failure symptoms of New-York-Heart-Association (NYHA) class IV (28), elevated levels of NTproBNP > 5,000 µg/L (21) and secondary organ failure, e.g., occurrence of cardiohepatic syndrome (29), were also associated with reduced survival. Coronary artery disease (CAD), in contrast, was not found to be an independent predictor of mortality, but the presence of the disease is discussed as a surrogate parameter of increased morbidity, in which other diseases may be prevalent, which in turn negatively influence the all-cause mortality rate (30).

In addition to cardiac comorbidities, other complex diseases were also relevant in influencing mortality. A study by Tabata et al. demonstrated significantly higher inflammation parameters and worse survival among patients who underwent M-TEER and had a history of cancer (31). However, further studies are needed to clarify to what extent the negative influence can be attributed to the disease itself or possibly to the utilization of potentially cardiotoxic chemotherapy regimens. Frailty or a low body mass index (BMI) were also linked to increased procedural risk and, consequently, increased mortality (32). Last, previous valve surgery was found to be an operative factor associated with increased mortality in this interventional cohort (21, 28).

#### Risk scores for the stratification of mortality prior to M-TEER

Many of the aforementioned predictors were then incorporated with other parameters to develop corresponding risk scores. The European System for Cardiac Operative Risk Evaluation (euroSCORE) II and the Society of Thoracic Surgeons' Predicted Risk of Mortality (STS-PROM) score represent the most well-known and earliest risk scores for predicting mortality and were originally developed for patients undergoing cardiac surgery (33, 34). While the EuroSCORE II inquires a smaller number of parameters and estimates all-cause mortality (AUC-value 0.81 for patients undergoing cardiac surgery), the STS-PROM score offers additional risk parameters in addition to mortality, e.g., renal failure, prolonged intensive care stay, permanent stroke and the need for reoperation (c-statistics 0.799 for mortality and c-statistics 0.639 for reoperation in patients undergoing cardiac surgery, respectively). Their validity for predicting mortality was subsequently confirmed equally for patients who underwent M-TEER, enabling the utilization of both scores in clinical risk prediction (35). An analysis by Schneider et al. also revealed an inverse association between the STS-PROM score and the procedural success of M-TEER, as measured by the reduction success of MR to mild severity (36).

Nevertheless, both scores were supplemented by further scores during the ongoing M-TEER treatment period. The MitraScore predicts the mortality and hospitalization rate after M-TEER on the basis of eight clinical points (c-statistics 0.68), which was also validated by various analyses (37, 38). The score was later expanded by adding the TAPSE/PASP-ratio as the aforementioned marker of RV-PA uncoupling (39). The MITRALITY score was also developed on the basis of clinical parameters for risk stratification prior to M-TEER and demonstrated comparable precision to the previously established scores in subsequent analyses (37, 40) (AUC value 0.783). The Mitral Regurgitation International Database (MIDA) risk score (41) (no AUC value or c-statistics provided) and the Getting Reduction of mitrAl inSufficiency (GRASP) nomogram (42) were equally developed on the basis of clinical parameters (AUC value 0.78). The MitraCox score stratifies the risk of in-hospital mortality and includes an evaluation of the conducting cardiac center with respect to the annual number of performed procedures (43) (AUC value 0.82). In contrast, the Cardiovascular Outcomes Assessment of the Mitraclip Percutaneous Therapy for Heart Failure Patients with Functional Mitral Regurgitation (COAPT) risk score (44) focuses on the risk assessment of patients with functional MR who meet the inclusion criteria of the COAPT study (8) (AUC value 0.74). All in all, the risk scores available to date are primarily predictive of mortality among M-TEER patients. To our knowledge, the STS-PROM score is the only score that can predict the additional endpoints listed. The previously published scores for the prediction of mortality risk in M-TEER patients are listed in Table 1.

**Table 1:** Overview of currently published scores for predicting mortality in patients undergoing M-TEER.

Score	Included variables	Outcome parameter	Precision^a^
**euroSCORE II** (33)	- Age - Sex - Chronic lung disease - Peripheral arterial disease - Impairment of mobility - Previous cardiac surgery - Active endocarditis - Hemodynamic or respiratory instability - Renal impairment - Diabetes mellitus requiring insulin - CCS angina class IV - LV function - Pulmonary hypertension - NYHA class - Planned surgery of the aorta - Urgency of the operation - Interventions that require the opening of the pericardium	All-cause mortality	AUC-value (patients undergoing cardiac surgery): 0.81 (33)
**STS-PROM score** (34)	- Surgery incidence & priority, concomitant TV repair - Sex - Age - Weight &Height - Race - Health insurance status - Creatinine - Hematocrit, WBC count, platelet count - Preoperative medication (ACE inhibitors/ARB, GP IIb/IIIa inhibitors, inotropes, steroids, ADP inhibitors) - Comorbidities (Diabetes mellitus, family history of CAD, arterial hypertension, liver disease, prior mediastinal radiation, prior unresponsive state/syncope, dialysis, cancer ≤ 5 years prior to surgery, immunocompromised status, endocarditis, illicit drug use, alcohol/tobacco use, chronic lung disease including sleep apnea and home oxygen therapy, recent pneumonia, cerebrovascular and peripheral artery disease, prior carotid surgery) - Cardiac status (type of heart failure, NYHA class, preoperative mechanic circulatory support, ejection fraction, primary coronary symptoms, time of myocardial infarction, number of diseased coronary arteries - Valve disease (aortic stenosis/regurgitation, mitral stenosis/regurgitation, tricuspid regurgitation, aortic root abscess - Arrhythmia (AF, AFL, VF, Vfib, SSS, AV-Block)	Operative mortality Permanent stroke Renal failure Prolonged ventilation (> 24 h) Deep sternal wound infection Reoperation for any reason, Major morbidity or mortality composite endpoint Prolonged postoperative length of stay Short postoperative length of stay	c-statistic (mortality in patients undergoing cardiac surgery) 0.799 (34) c-statistic (probability of re-operation in patients undergoing cardiac surgery) 0.639 (34)
**MitraScore** (38)	- Age ≥ 75 years - LVEF < 40% - Anemia - eGFR <60 ml/min/1.73 m^2^ - Peripheral artery disease - COPD - High dose of diuretics - No therapy with RAS inhibitors	All-cause mortality Rehospitalization for heart failure Probability of clinical improvement	c-statistic (M-TEER patients) 0.68
TAPSE/PASP MitraScore (39)	- TAPSE/PASP-ratio < 0.37 mm/mmHg additional to the variables of the MitraScore (38)	All-cause mortality Rehospitalization for heart failure	NA
MITRALITY score (40)	- Urea - Hemoglobin - NTproBNP - Mean arterial pressure - Body mass index - Creatinine	All-cause mortality 1 year after M-TEER	AUC-value (M-TEER patients) 0.783 (40)
**MIDA Risk score** (41)	- Age ≥ 65 years - Heart failure symptoms - AF - Left atrial diameter ≥ 55 mm - Right ventricular systolic pressure > 55 mmHg - Left ventricular endsystolic diameter ≥ 40 mm - LVEF ≤ 60%	Composite endpoint of all-cause mortality and rehospitalization for heart failure 2 years after M-TEER	NA
MitraCox score (43)	- Age - Admission in a teaching hospital - Location of hospital - Number of M-TEER procedures per year - Comorbidities (arterial/pulmonary hypertension, congestive heart failure, coagulation disturbances, liver disease except cirrhosis, drug abuse, tobacco use, psychosis, hypothyroidism, malnutrition, fluid/electrolyt disturbances, Impella insertion, prior balloon angioplasty, bare metal stent in-situ)	In-hospital mortality	c-statistic (M-TEER patients) 0.82
GRASP score (42)	- Mean arterial pressure - Hemoglobin - NTproBNP - NYHA class IV	All-cause mortality 1 year after M-TEER	AUC-value (M-TEER patients) 0.78 (42)
**COAPT risk score** (44)	- COPD - NYHA class III/IV - AF/AFL - Chronic kidney disease stage III/IV - LVEF < 35% - Right ventricular systolic pressure > 45 mmHg - Left ventricular endsystolic diameter > 55 mm - - > Mild tricuspid valve regurgitation	Composite endpoint of all-cause mortality and rehospitalization for heart failure 2 years in patients with functional MR that meet the COAPT inclusion criteria (8) after M-TEER	AUC-value (M-TEER patients) 0.74

a The precision of the score in estimating mortality was reported either as an AUC-value or as a correlation coefficient (c-statistic) according to the reported findings in the cited study. Other reported parameters were classified as “NA“.

ACE, angiotensin-converting-enzyme; ADP, adenosine diphosphate; AF, atrial fibrillation; AFL, atrial flutter; ARB, angiotensin receptor blocker; AUC, area under the curve; AV, atrioventricular; CAD, coronary artery disease; CCS, canadian cardiovascular society; COAPT, cardiovascular outcomes assessment of the mitraclip percutaneous therapy for heart failure patients with functional mitral regurgitation; COPD, chronic obstructive pulmonary disease; eGFR, estimated glomerular filtration rate; euroSCORE, european system for cardiac operative risk evaluation; GP, glycoprotein; GRASP, getting reduction of mitrAl inSufficiency; LV, left ventricle; LVEF, left ventricular ejection fraction; MIDA, mitral regurgitation international database risk score; M-TEER, transcatheter edge-to-edge mitral valve repair; MV, mitral valve; NA, not applicable; NYHA, New-York-Heart-Association; PASP, pulmonary artery systolic pressure; RAS, renin-angiotensin-system; SSS, sick sinus syndrome; STS-PROM, society of thoracic surgeons predicted risk of mortality; TAPSE, tricuspid annular plane systolic excursion; TV, tricuspid valve; VF, ventricular flutter; Vfib, ventricular fibrillation; WBC, white blood cell.

### Transcatheter edge-to-edge tricuspid valve repair (T-TEER)

#### Clinical parameters with significant effects on mortality after T-TEER

Since the first feasibility study on T-TEER was published ten years after the food and drug administration (FDA) approval of M-TEER and therefore represents a more recent form of treatment (6), the corresponding evidence with respect to mortality predictors is relatively limited. Similar to the M-TEER collective, concomitant pulmonary hypertension (45), RV-PA uncoupling (46, 47) and cardiohepatic and cardiorenal syndrome (48, 49) negatively impacted survival in patients who underwent T-TEER. The same applied to underweight (50) and malnutrition (51), which can also be regarded as markers of advanced cardiac and noncardiac morbidity. In this context, geriatric multimorbidity has emerged as a marker of reduced survival (52). Owing to the ongoing demographic changes, this factor occupies a relevant position in both TEER cohorts. In addition to the general risk assessment, the question arises as to whether geriatric multimorbidity can be optimized, which might have an impact on treatment outcomes. Thus, the conditions and effects of this parameter in both TEER collectives should be examined in more detail in future studies.

### Risk scores for the stratification of mortality prior to T-TEER

With respect to the risk scores for the prediction of mortality in the T-TEER cohort, the EuroSCORE II (AUC-value 0.81 for patients undergoing cardiac surgery) and the STS-PROM score (c-statistics 0.799 for mortality and c-statistics 0.639 for reoperation in patients undergoing cardiac surgery, respectively) can also be applied, but no dedicated validation of the scores was performed in this specific cohort. However, they can still provide a risk assessment for isolated surgical tricuspid valve repair, although this procedure is rarely performed because of the elevated complication rate in addition to the increased average morbidity of the respective cohort (53). The additional predicted endpoints of the STS-PROM score could be applied equally in the T-TEER cohort, although differentiated validation studies of the score in these endpoints among this particular cohort are currently lacking. In contrast, the TRI-SCORE outperforms the EuroSCORE II and STS-PROM score in the prediction of 30-day, 1-year and 10-year mortality after T-TEER (54, 55) (e.g., AUC values for predictin 1-year mortality after T-TEER: 0.931 vs. 0.644 vs. 0.59, respectively). An additional score also composed of clinical and echocardiographic parameters is the TRIVALVE score, which predicts a combined endpoint of mortality and rehospitalization until one year after T-TEER (56) (AUC-value: 0.683). Furthermore, the Get With The Guidelines-Heart Failure (GWTG-HF) score was extrapolated to T-TEER patients after it was developed primarily to predict in-hospital mortality in patients hospitalized due to acute heart failure (57). In a published study by Kavsur et al., the GWTG-HF score proved to be equally predictive of the combined endpoint of mortality and rehospitalization until one year after T-TEER (57) (no AUC value or c-statistics reported). Finally, the GLIDE score was developed to predict procedural success prior to T-TEER, defined as the reduction in TR to a moderate or lower severity grade (58, 59). This score was not intended to directly predict mortality but could be considered metaphorically, as procedural T-TEER success has been shown to be a marker of reduced mortality and hospitalization rates (60). An overview of the currently available scores that predict mortality after T-TEER is outlined in Table 2.

**Table 2:** Overview of currently published scores for predicting mortality in patients undergoing T-TEER.

Score	Included variables	Outcome parameter	Precision^a^
euroSCORE II (33)	- Age - Sex - Chronic lung disease - Peripheral arterial disease - Impairment of mobility - Previous cardiac surgery - Active endocarditis - Hemodynamic or respiratory instability - Renal impairment - Diabetes mellitus requiring insulin - CCS angina class IV - LV function - Pulmonary hypertension - NYHA class - Planned surgery of the aorta - Urgency of the operation - Interventions that require the opening of the pericardium	All-cause mortality	AUC-value (patients undergoing cardiac surgery): 0.81 (33) AUC-value (1-year-mortality rate, T-TEER patients): 0.644 (55) AUC-value (combined endpoint of mortality and hospitalization for heart failure 1 year after T-TEER): 0.566 (56)
STS-PROM score (34)	- Surgery incidence & priority, concomitant TV repair - Sex - Age - Weight &Height - Race - Health insurance status - Creatinine - Hematocrit, WBC count, platelet count - Preoperative medication (ACE inhibitors/ARB, GP IIb/IIIa inhibitors, inotropes, steroids, ADP inhibitors) - Comorbidities (Diabetes mellitus, family history of CAD, arterial hypertension, liver disease, prior mediastinal radiation, prior unresponsive state/syncope, dialysis, cancer ≤ 5 years prior to surgery, immunocompromised status, endocarditis, illicit drug use, alcohol/tobacco use, chronic lung disease including sleep apnea and home oxygen therapy, recent pneumonia, cerebrovascular and peripheral artery disease, prior carotid surgery) - Cardiac status (type of heart failure, NYHA class, preoperative mechanic circulatory support, ejection fraction, primary coronary symptoms, time of myocardial infarction, number of diseased coronary arteries - Valve disease (aortic stenosis/regurgitation, mitral stenosis/regurgitation, tricuspid regurgitation, aortic root abscess - Arrhythmia (AF, AFL, VF, Vfib, SSS, AV-Block)	Operative mortality Permanent stroke Renal failure Prolonged ventilation (> 24 h) Deep sternal wound infection Reoperation for any reason, Major morbidity or mortality composite endpoint Prolonged postoperative length of stay Short postoperative length of stay	c-statistic (mortality in patients undergoing cardiac surgery) 0.799 (34) c-statistic (probability of re-operation in patients undergoing cardiac surgery) 0.639 (34) AUC-value (1-year-mortality rate, T-TEER patients): 0.59 (55) AUC-value (combined endpoint of mortality and hospitalization for heart failure 1 year after T-TEER): 0.611 (56)
TRI-SCORE (54, 55)	- Age ≥ 70 years - NYHA class III/IV - Right-sided heart failure - Daily dose of furosemide ≥ 125 mg - eGFR < 30 ml/min - Elevated bilirubin - LVEF < 60% - Moderate/severe RVD	All-cause mortality 30 days, 1 year, 10 years after T-TEER	AUC-value (30 days mortality rate, T-TEER patients): 0.903 (55) AUC-value (1-year mortality rate, T-TEER patients): 0.931 (55) AUC-value (10-year-mortality rate, T-TEER patients): 0.83 (54)
TRIVALVE score (56)	- AF - eGFR < 30 ml/min - Elevated GGT/bilirubin - Right-sided heart failure - LVEF < 50%	Composite endpoint of all-cause mortality and rehospitalization for heart failure 1 year after T-TEER	AUC-value (T-TEER patients): 0.681
GWTG-HF risk score (57)	- Age - Race - Heart rate - Urea - Sodium - COPD	Composite endpoint of all-cause mortality and rehospitalization for heart failure 1 year after T-TEER	NA

a The precision of the score in estimating mortality was reported either as an AUC-value or as a correlation coefficient (c-statistic) according to the reported findings in the cited study. Other reported parameters were classified as “NA“.

ACE, angiotensin-converting-enzyme; ADP, adenosine diphosphate; AF, atrial fibirllation; AFL, atrial flutter; ARB, angiotensin receptor blocker; AUC, area under the curve; AV, atrioventricular; CAD, coronary artery disease; CCS - canadian cardiovascular society; COPD, chronic obstructive pulmonary disease; eGFR, estimated glomerular filtration rate; euroSCORE, european system for cardiac operative risk evaluation; GGT, gamma-glutamyl transferase; GP, glycoprotein; GWTG-HF, get with the guidelines-heart failure risk; LV, left ventricle; LVEF, left ventricular ejection fraction; NA, not applicable; NYHA, New-York-Heart-Association; RVD, right ventricular dysfunction; SSS, sick sinus syndrome; STS-PROM, society of thoracic surgeons predicted risk of mortality; T-TEER, transcatheter edge-to-edge tricuspid valve repair; TV, tricuspid valve; VF, ventricular flutter; Vfib, ventricular fibrillation; WBC, white blood cell.

In summary, previous research revealed a broad spectrum of cofactors that were independently associated with increased mortality following M-TEER and T-TEER. To the best of our knowledge, current evidence does not indicate a relevant influence of the choice of TEER system on mortality (61, 62). While favorable results have been reported for the newer M-TEER devices in terms of procedure-related characteristics and hospitalization rates, no results are yet available for mortality (63). However, some study findings showed that mortality can be significantly ameliorated by treatment of the underlying comorbidity or by TEER itself. For instance, the survival of patients who underwent M-TEER and had concomitant AF could be considerably improved by pulmonary vein isolation as a state-of-the-art therapy for AF to the extent that survival no longer differed from that of patients who underwent M-TEER without AF (64). Similarly, an improvement in RV-PA uncoupling after M-TEER was observed in the majority of patients, which was associated with improved survival after M-TEER (65). With respect to secondary organ failure due to advanced VHD, T-TEER led to an improvement in liver function, while renal function remained unaffected (66).

These recent data raise the question as to what extent the appropriate treatment of the underlying condition and the TEER procedure can contribute to improved survival, thereby justifying the referral of the patient for TEER. The available preliminary findings provide a significant contribution to existing evidence, which should, however, be further explored and augmented by future studies.

While the STS-PROM score represents the highest versatility in predicted endpoints among M-TEER patients (34), it can be supplemented by the MITRALITY score with the best AUC value to date (40), although comparability is hampered by inconsistencies in the reports. Among T-TEER patients, the TRI-SCORE has so far proven to be even better at predicting mortality than the well-known euroSCORE II and STS-PROM score models (54, 55). For future treatment, risk prediction should therefore be based on the identified comorbidities and a selection of the aforementioned scores. Nevertheless, depending on the results of upcoming studies, further scores should be developed that take into account any prognosis-improving therapies for comorbidities. In addition, other endpoints besides mortality, such as the occurrence of procedural complications and the reduction success of MR/TR, should also be investigated as further crucial endpoint parameters.

### Limitations

Given that this review did not aim to serve as a systematic review, the effects of additional unknown or uncollected cofactors on mortality cannot be excluded. The respective limitations that were outlined in the particular studies should be considered when the presented findings are interpreted. Notably, individual confirmation of the feasibility of M-TEER by echocardiography constitutes the cornerstone of further risk assessment prior to the procedure. This review did not include a synthesis of the relevant echocardiographic feasibility criteria, therefore these should be considered separately in the relevant publications (67, 68). On the basis of the present analysis, no statement can be drawn regarding the risk prediction of patients undergoing transcatheter mitral and tricuspid valve replacement (TMVR, TTVR), who therefore must also be examined separately.

## Conclusion

Clinical decision-making in the current TEER era remains challenging but can be supported by a variety of cofactors that are linked to survival and by various risk scores that estimate all-cause mortality. Although none of these parameters can provide the sole criterion for deciding for or against TEER, the presented overview can significantly contribute to optimized patient selection with consecutive improvement in treatment outcomes. Further research is needed to investigate the effects of the treatment of comorbidities that significantly worsen mortality.

## Glossary

ACE: angiotensin-converting-enzyme
ADP: adenosine diphosphate
AF: atrial fibrillation
AFL: atrial flutter
ARB: angiotensin receptor blocker
AUC: area under the curve
AV: atrioventricular
BMI: body mass index
CAD: coronary artery disease
CCS: canadian cardiovascular society
COAPT risk score: cardiovascular outcomes assessment of the mitraclip percutaneous therapy for heart failure patients with functional mitral regurgitation risk score
COPD: chronic obstructive pulmonary disease
eGFR: estimated glomerular filtration rate
euroSCORE II: european system for cardiac operative risk evaluation II
FDA: food and drug administration
GGT: gamma-glutamyl transferase
GP: glycoprotein
GRASP normogram: getting reduction of mitrAl inSufficiency normogram
GWTG-HF score: get with the guidelines-heart failure risk score
LV: left ventricle
LVEF: left ventricular ejection fraction
MIDA risk score: mitral regurgitation international database risk score
MR: mitral valve regurgitation
M-TEER: transcatheter edge-to-edge mitral valve repair
MV: mitral valve
NA: not applicable
NYHA: New-York-Heart-Association
OMT: optimal medical therapy
PA: pulmonary artery
PASP: pulmonary arterial systolic pressure
RAS: renin-angiotensin-system
RV: right ventricle
RVD: right ventricular dysfunction
SSS: sick sinus syndrome
STS-PROM score: society of thoracic surgeons predicted risk of mortality score
TAPSE: tricuspid annular plane systolic excursion
TMVR: transcatheter mitral valve replacement
TR: tricuspid valve regurgitation
T-TEER: transcatheter edge-to-edge tricuspid valve repair
TTVR: transcatheter tricuspid valve replacement
TV: tricuspid valve
VF: ventricular flutter
Vfib: ventricular fibrillation
VHD: valvular heart disease
WBC: white blood cell
